# Development of Core Contents of Point-of-Care Ultrasound Curriculum for Pediatric Emergency Medicine Physician Training: A Modified Delphi Survey

**DOI:** 10.3390/children8090757

**Published:** 2021-08-31

**Authors:** Jae-Hyun Kwon, Jin-Hee Lee, Young-Rock Ha, June-Dong Park, on behalf of the Division of Pediatric Emergency Ultrasound, Korean Society of Pediatric Emergency Medicine and Society of Emergency & Critical Care Imaging

**Affiliations:** 1 Department of Emergency Medicine, Bundang CHA Hospital, CHA University School of Medicine, Pocheon 13496, Korea; rirengarnat@hotmail.com; 2 Department of Emergency Medicine, Seoul National University Bundang Hospital, Seoul National University College of Medicine, Seoul 13620, Korea; 3 Department of Critical Care Medicine, Seongnam Citizens Medical Center, Seongnam 13290, Korea; rocky66@naver.com; 4 Department of Pediatrics, Seoul National University College of Medicine, Seoul 03080, Korea; jdparkmd@snu.ac.kr

**Keywords:** ultrasound, pediatrics, pediatric emergency medicine, education

## Abstract

Background: As the frequency of ultrasound use in pediatric emergency departments increases, it is necessary to train pediatric emergency medicine (PEM) physicians on pediatric point-of-care ultrasonography (POCUS). We discussed the core content of POCUS applications and proposed a POCUS training curriculum for PEM physicians in South Korea. Methods: Twenty-three experts were included if had performed over 1500 POCUS scans, had at least three years of experience teaching POCUS to physicians, were POCUS instructors or had completed a certified pediatric POCUS program. Experts rated 61 possible POCUS applications in terms of the importance of their inclusion in a PEM POCUS curriculum using the modified Delphi technique. Results: In round one, twelve (52.2%) out of 23 experts responded to the email. Eleven experts satisfied the inclusion criteria. Eleven experts participated in round one of a survey and agreed on 27 (44.3%) out of a total of 61 items. In round two, all 11 experts participated in the survey; they agreed on two (5.9%) of the remaining 34 items, and no items were excluded. Conclusion: Using the Delphi method, 61 applications were discussed, and a consensus was reached on 29 core applications.

## 1. Introduction

Every year, approximately 10 million patients visit emergency departments (EDs) in Korea [1], and according to the National Emergency Department Information System (NEDIS) statistics report, patients under 19 years old accounted for 24.4% of the patients who visited 153 EDs at the center level or higher in the first half of 2018 [2].

The number of children visiting EDs is increasing every year [3], and the nature and frequency of disease or trauma in these patients is different from that in pediatric outpatients [4]. In Korea, a pediatrician or an emergency medicine specialist who has completed a PEM fellowship works as a PEM physician. Patients visiting the ED have conditions of varying severity, so rapid assessment and treatment based on the level of severity are required, especially for trauma patients. Due to the risk of radiation exposure inherent in the use of X-ray and computed tomography (CT), which increases onset of leukemia or cancer in children, the use of pediatric point-of-care ultrasound (POCUS) in the ED is important for achieving rapid assessment and diagnosis [5]. The characteristics of pediatric diseases to be identified vary according to age and symptoms, so the application of POCUS to pediatric patients should include diagnoses that do not occur in adult patients (such as hypertrophic pyloric stenosis and intussusception).

In the United States, clinical guidelines are presented for PEM physicians in the 2015 American Academy of Pediatrics (AAP), American College of Emergency Physicians (ACEP), Society for Academic Emergency Medicine (SAEM), and World Interactive Network Focused on Critical Ultrasound (WINFOCUS), and POCUS training is necessary for pediatric physicians to be licensed [6,7,8]. POCUS is essential for the education of dedicated PEM physicians, and a core POCUS education has been recommended for the training of PEM physicians at the Royal College of Physicians and Surgeons of Canada [9,10].

In Korea, in 2016, the Korean Society of Pediatric Emergency Medicine (KSPEM) and Society of Emergency & Critical Care Imaging (SECCI) partnered and established the Division of Pediatric Emergency Ultrasound Education. With their own experience and a consensus regarding internal minorities, they began to create and proceed with a pediatric POCUS education program. However, as the number of pediatric emergency specialists increased and pediatric POCUS experience accumulated, it became necessary to investigate expert consensus on the educational curriculum of pediatric POCUS for more systematic education. In Korea, there may be differences from the United States and Canada with regard to the medical system, insurance, and educational environment, so there is a need to develop an educational curriculum suitable for the Korean environment. This study aimed to select the core contents of educational programs for POCUS and suggest the direction of future Korean PEM POCUS education through expert Delphi consultation.

## 2. Materials and Methods

This study was reviewed and exempted from deliberation by the Institutional Review Board of Bundang CHA Hospital (IRB No. 2019-10-069. 15 November 2019). Informed consent was obtained from all participants in Delphi.

### 2.1. Characteristics of the Expert Group

To develop representative and professional recommendations, a total of 23 panels were commissioned by the KSPEM and SECCI. To protect the privacy and confidentiality of individuals during recruitment, individuals filled out the questionnaire only upon agreeing to participate in the study after the research was explained, and were excluded from the study upon not agreeing to participate.

### 2.2. Experts

The criteria for the experts were as follows: (1) more than 3 years of experience in training specialists on POCUS related to pediatric emergencies; (2) currently serving as an instructor of a pediatric emergency POCUS course and (3) completed KSPEM-sponsored pediatric emergency POCUS course training. Those who met at least one of the above three criteria, and had experience with at least 1500 cases of POCUS scans, were selected [11]. Those who did not meet the above criteria were excluded from the study.

### 2.3. Study Design

The modified Delphi method is a research method that allows an expert group to reach a consensus through two or more questionnaires. The number of people who can reach a consensus in Delphi research typically ranges from 4 to 200 [12,13,14,15,16]. We determined items that are likely to be included in the POCUS training curriculum for pediatric emergency physicians through literature reviews. A questionnaire was developed by selecting a total of 61 items among POCUS applications mainly used for training in the United States and the United Kingdom, especially ACEP POCUS applications. After the questionnaire was developed, it was completed after review by the board of directors of the POCUS expert group.

The applications list was selected after literature review [17,18] and table discussions with the authors of this study when selecting the list of application surveys. The questionnaire prepared in this way was intended to be a new educational guideline for PEM POCUS by adopting the agreed-upon educational plan after a total of two rounds of Delphi agreement. The modified Delphi method was used as a consensus-based method for deriving and adopting recommendations. To help experts decide, the questionnaire was filled out regarding the importance of each technique by providing Delphi panelist references by email. We received new statements/suggestions at the end of the survey. In the questionnaire there were 10 subtitles in three areas, for a total of 61 items. Experts who received the questionnaire were asked to score a scale of 1 to 9. When analyzing the questionnaire for each item, a score of 1 to 3 points was classified as “inappropriate”, a score of 4 to 6 points was classified as “unclear”, and a score of 7 to 9 points was classified as “recommended”.

A consensus should not be forced, and the outcome could lead to a classification of “appropriate”, “uncertain”, or “inappropriate”. Therefore, the degree of agreement on the recommendation was quantified on a 9-point Likert scale, and when the result showed a total score of 7 points or more for 70% or more of the experts, it was decided that the recommendation was agreed upon [19,20,21,22]. In the second round, the median score of all experts in the previous round, and the score of the experts who answered the questionnaire, were provided, and the questionnaire was conducted excluding items that had been agreed upon in the previous round. For items that failed to reach a consensus in the previous round, amendments were made by synthesizing other opinions presented by panelists. The intent was that panelists’ opinions could change the relevant application to include more specific content. As in Round 1, the questionnaire collected in round 2 was classified on a 9-point Likert scale.

### 2.4. Survey

The pediatric POCUS applications were selected for inclusion in the Delphi process by searching the current literature and reaching a consensus [18,23]. All members of the research team reviewed and edited each iteration of the survey, and pilot testing was used to identify errors and corrections.

### 2.5. Statistical Analysis

All statistical analyses were performed using the SPSS statistical package (version 22, IBM Corp., Armonk, NY, USA). Continuous variables are presented as the mean (SD) or median (interquartile range; IQR). Categorical variables are described with frequencies and proportions (%).

## 3. Results

### 3.1. General Characteristics

We asked 23 panelists recommended by the KSPEM and SECCI to participate in the survey by email. A total of 12 experts (52.2%) responded to the first survey and a total of 11 (91.7%) were included; one (8.3%) was not included in the expert group based on the predefined criteria. All 11 experts (100%) responded to the second survey. Participants who met the inclusion criteria had an average of 9.6 years (9.0, 4.0–15.0) of experience. Among the specialties, emergency medicine was the most frequent (64%) (Table 1).

### 3.2. Inclusion of Core Applications

It was recommended that a consensus be reached by more than 70% of participants scoring an item at least seven points, and a consensus was reached for 29 of 61 items after two Delphi rounds (Table 2), as follows: resuscitative applications (identify free peritoneal fluid, identify pericardial effusion, identify pneumothorax, identify hemothorax, evaluate cardiac function, identify cardiac standstill, identify nontraumatic pericardial effusion, identify myocarditis, identify pulmonary edema); diagnostic applications (identify pyloric stenosis, identify intussusception, identify mesenteric adenitis, identify appendicitis, identify hydronephrosis, identify testicular torsion, identify epididymo-orchitis, identify abscess, identify soft tissue foreign body, identify joint effusions, identify cellulitis, identify bone fracture), and POCUS-assisted/guided procedural applications (arterial line placement, central line placement, foreign body localization and removal, pericardiocentesis, arthrocentesis, pleurocentesis, paracentesis, suprapubic bladder aspiration). The 29 applications of POCUS recommended for inclusion in training by a dedicated PEM physician are presented in Table 3. If a score of three or less reached an agreement of 70% or more, it was decided that the recommendation was inappropriate and should be excluded. As a result of the Delphi method, none of the 61 items were classified as inappropriate (Table 2). The remaining 32 items were not agreed upon for recommendation or exclusion; these 32 items are shown in Table 4.

## 4. Discussion

Improving the competence of PEM physicians through systematic and professional POCUS training is essential for the accurate diagnosis and treatment. In addition, there is a need for a PEM POCUS-related relationship that provides physicians with the management, research and academic skills necessary to create a POCUS program and train future POCUS leaders. Through such a relationship, physicians with POCUS training will be provided with greater opportunities through workshops, immersive training and trainee mentoring.

In 2016 in the U.S., ACEP updated their emergency, point-of-care, and clinical ultrasound guidelines. Evidence-based POCUS applications in the ED were added annually. In 2019, 48 out of 60 applications were agreed upon in discussions of POCUS applications and were recommended for inclusion in the educational curriculum for PEM fellows [18]. The Royal College of Physicians and Surgeons of Canada began to incorporate mandatory training in POCUS into PEM fellowship training curricula to match current evidence [9,10].

In South Korea, SECCI was founded in 2014 to promote academic discussion and improve emergency POCUS and imaging skills. Over the past 5 years, SECCI has held many POCUS workshops and conferences. Guidelines for the use of POCUS in the ED in Korea were published in 2020 [24]. Accordingly, there is a need to reach an expert consensus on pediatric POCUS education, which was the motivation for this study.

The following Korean PEM POCUS applications were the same as those in the suggested PEM POCUS curriculum presented at the CAEP annual conference: identify free peritoneal fluid, identify pericardial effusion, identify pneumothorax, identify hemothorax, evaluate cardiac function, identify cardiac standstill, identify nontraumatic pericardial effusion, identify pulmonary edema, identify intussusception, identify abscess, identify soft tissue foreign body, identify cellulitis, place a central line, localize and remove a foreign body, and perform pericardiocentesis. There was no consensus regarding whether to include the following applications in the PEM POCUS curriculum: identify pyloric stenosis, identify mesenteric adenitis, identify appendicitis, identify hydronephrosis, identify testicular torsion, identify joint effusions, identify bone fracture, place an arterial line, perform arthrocentesis, perform pleurocentesis, perform paracentesis, and perform suprapubic bladder aspiration [18]. There were differences in the applications included in the two curriculums. These differences may be due to the interests of the participants in each study, which would affect what they consider important, the differences in the medical systems in the two countries, and whether radiologists are always available. In many hospitals in Korea, radiologists are not available at night or on holidays, so there are many hospitals that need to consider using CT to diagnose pediatric patients.

We suggest that the 29 POCUS applications agreed upon in this study should be included in PEM POCUS educational programs; however, because of lack of consensus, it is expected that correction and supplementation will be needed through additional investigations in the future.

The limitations of this study are as follows. First, the number of participants in the Delphi consultation was small. The survey was conducted via email, but the response rate was approximately 50%. However, the number of people required for consultation is generally known to range from 4 to 200 [12,13,14,15,16], and as all participants were experts, the outcome of the consultation would not be affected. Second, the experts did not consider any of the items to be inappropriate. Therefore, even items for which agreement was not reached may need to be taught, but there are difficulties implementing and applying the training. This suggests that further investigation may be required after the training of instructors and PEM doctors in the field, as well as in POCUS workshops. Third, we investigated the POCUS guideline only written in English. Although these applications are used worldwide, there is a limit to finding better suggestions in other language.

## 5. Conclusions

It is important to determine the pediatric POCUS training curriculum for diagnostic screening and procedural POCUS programs. Using the Delphi method, 61 applications were discussed and a consensus was reached on 29 core applications.

## Figures and Tables

**Table 1:** Profile of Expert Respondents Completing Round 1 (*n* = 11).

Characteristic	No. (%)
Years of practice outside training, median (IQR)	9.0 (4.0–15.0)
Postgraduate training	
Pediatrics	3 (27)
Emergency medicine	7 (64)
Pediatrics and Emergency medicine	1 (9)
Inclusion criteria	
PEM POCUS course trainer over 3 years	8 (73)
PEM POCUS trainer or director at present	7 (64)
PEM POCUS course completed	8 (73)
Over 1500 clinical POCUS exams	11 (100)

IQR = interquartile range; PEM = pediatric emergency medicine; POCUS = point-of-care ultrasound.

**Table 2:** Number of POCUS Applications by Delphi Round.

	Round 1 (61 Applications)	Round 2 (34 Applications)	Final Applications
Included	27 (44.3)	2 (5.9)	29
Excluded	0 (0)	0 (0)	0
No consensus	34 (55.7)	32 (94.1)	32

Data are reported as *n* (%). POCUS = point-of-care ultrasound.

**Table 3:** POCUS Applications to Include by Delphi Technique.

Round	Application	No. of Experts Who Ranked Item as Very Important (%)
Round1		
	Resuscitative applications	
	Identify free peritoneal fluid	11 (100)
	Identify pericardial effusion	11 (100)
	Identify pneumothorax	8 (73)
	Identify hemothorax	9 (82)
	Evaluate cardiac function	11 (100)
	Identify cardiac standstill	9 (82)
	Identify nontraumatic pericardial effusion	9 (82)
	Identify myocarditis	8 (73)
	Identify pulmonary edema	8 (73)
	Diagnostic applications	
	Identify pyloric stenosis	10 (91)
	Identify intussusception	11 (100)
	Identify mesenteric adenitis	9 (82)
	Identify appendicitis	11 (100)
	Identify hydronephrosis	10 (91)
	Identify testicular torsion	11 (100)
	Identify epididymo-orchitis	9 (82)
	Identify abscess	9 (82)
	Identify soft tissue foreign body	10 (91)
	Identify joint effusion	9 (82)
	POCUS-assisted/guided procedural applications	
	Arterial line placement	8 (73)
	Central line placement	11 (100)
	Foreign body localization and removal	8 (73)
	Pericardiocentesis	9 (82)
	Arthrocentesis	9 (82)
	Pleurocentesis	9 (82)
	Paracentesis	9 (82)
	Suprapubic bladder aspiration	9 (82)
Round2		
	Diagnostic applications	
	Identify cellulitis	8 (73)
	Identify bone fracture	8 (73)

Data are reported as *n* (%). POCUS = point-of-care ultrasound.

**Table 4:** POCUS Applications with No Consensus to Include or Exclude by Delphi Technique.

Application	No. of Experts Who Ranked Item as Very Important (%)
Resuscitative applications	
Identify ET tube replacement	7 (64)
Identify lung consolidation	7 (64)
Assess IVC for volume status	5 (45)
Assess IVC-to-aorta ratio	2 (18)
Diagnostic applications	
Identify abdominal aortic aneurysm (AAA)	1 (9)
Assess bladder volume	3 (27)
Identify cholelithiasis	5 (45)
Identify cholecystitis	5 (45)
Assess for ovarian torsion	4 (36)
Identify adnexal abscess	2 (18)
Assess for ovarian cyst/mass	5 (45)
Identify intrauterine pregnancy	4 (36)
Determine viability of intrauterine pregnancy	1 (9)
Identify testicular mass	6 (55)
Identify necrotizing fasciitis	5 (45)
Identify myositis	3 (27)
Identify skull fracture	6 (55)
Identify peritonsillar abscess	2 (18)
Identify neck adenitis	4 (36)
Identify deep vein thrombosis	2 (18)
Identify retinal detachment	3 (27)
Identify vitreous hemorrhage	1 (9)
Identify vitreous detachment	1 (9)
Identify lens dislocation	2 (18)
Evaluate optic nerve for papilledema	5 (45)
POCUS-assisted/guided procedural applications	
Peripheral IV access	7 (64)
IO needle confirmation	6 (55)
Abscess incision and drainage	7 (64)
Lumbar puncture	5 (45)
Nerve block	1 (9)
Fracture reduction	0 (0)
Peritonsillar abscess drainage	0 (0)

Data are reported as *n* (%). POCUS = point-of-care ultrasound.

## Data Availability

Research data is available at https://drive.google.com/file/d/1nFImIYLkMlAL6z3UXI8JuQaA1bBwoluA/view?usp=sharing (accessed on 29 August 2021).
